# Community Pharmacists’ Responses Toward Antimicrobial Prescriptions in Jordan: A Cross-Sectional Survey

**DOI:** 10.3390/antibiotics14030300

**Published:** 2025-03-14

**Authors:** Ma’en Al-Odat, Shadi Mustafa, Yousef Al-Hajaya, Anwar Kandari, Amane Alaroud, Ahmad Alenezi, Haitham Qaralleh, Yasmeen Hazaimeh

**Affiliations:** 1 Department of Medical Laboratory Science, Faculty of Allied Medical Science, Mutah University, Karak 61710, Jordan; haitham@mutah.edu.jo; 2 Faculty of Pharmacy, Jerash University, Jerash 26150, Jordan; shadi.mustafa@outlook.com; 3 Department of Biological Sciences, Faculty of Science, Mutah University, Karak 61710, Jordan; yousef.hajaya@mutah.edu.jo; 4 Dasman Diabetes Institute, Kuwait City 15462, Kuwait; anwar.kandari@dasmaninstitute.org; 5 Faculty of Pharmacy, Mutah University, Karak 61710, Jordan; a.alaroud@mutah.edu.jo (A.A.); yasmeenahmad424@gmail.com (Y.H.); 6 Radiologic Sciences Department, Health Sciences Center, Kuwait University, Kuwait 12037, Kuwait

**Keywords:** antimicrobials, antimicrobial use, antibiotics, pharmacist, cross-sectional survey

## Abstract

**Background:** Globally, community pharmacists worldwide have prescribed more over-the-counter systemic antibiotics, posing significant issues for international organizations tackling antibiotic-resistant bacteria, a major global threat, due to the accessibility in pharmacies. **Objectives:** This study aimed to examine the Jordanian pharmacists’ antibiotic selection and over-the-counter antibiotic prescriptions. **Methods:** A total of 244 community pharmacists participated in an online standardized survey, which examined five essential domains including sources and trust of pharmacy antibiotic prescription information, category and frequency of permitted antimicrobials, prescription-free antimicrobials, interactions, antimicrobial prescription issues for pharmacists, and pharmacy staff’s knowledge of non-prescription antimicrobial questions and answers. **Results:** This study found that 1—pharmacists are confident in prescribing antibiotics and they use various tools to improve their skills. 2—Antibiotics were the most sought antimicrobials without a prescription, followed by antifungals and antivirals. 3—Throat, urinary tract, chest, and otitis media are the most common infections that require antibiotics. Pharmacists prescribe penicillin for 75% of throat infections, Fluoroquinolones for 48.2% of urinary tract infections, and cephalosporins for 35.9%. Macrolides are the most prevalent otitis media treatment (43.2%). 4—Some people obtain antimicrobial prescriptions without a valid reason or diagnosis. 5—Many pharmacists (171/244, 70%) agree or strongly believe that antibiotic prescription information is difficult to obtain. 6—Many pharmacists (183/200, 91.5%) aimed to educate patients on the risks and correct use of antimicrobials without prescriptions. **Conclusions:** These results show that Jordanian community pharmacists follow clinical antibiotic prescribing guidelines and conduct antimicrobial stewardship, yet they demand antimicrobials without prescriptions and lack decision support tools. Antibiotic classes address most diseases, and pharmacists emphasize antimicrobial misuse.

## 1. Introduction

Antimicrobial medications are pharmacological agents implemented in the treatment of infections induced by microorganisms, which encompass bacteria, viruses, fungi, and parasites. These agents operate through mechanisms that either eliminate pathogens or suppress their proliferation, thus facilitating the resolution of infections and mitigate their transmission. Their development and application have significantly diminished the morbidity and mortality linked to infectious diseases [1,2,3,4].

During the previous decade, over-the-counter systemic antibiotic prescriptions from community pharmacists have risen considerably internationally [5,6]. The antimicrobial stewardship protocols are recommended for proper antibiotic prescribing to reduce antimicrobial resistance in Jordan [7]. Consequently, ensuring patient access to antibiotics through pharmacies poses a significant challenge for international organizations striving to combat the widespread emergence of antibiotic-resistant mutations, which represent a serious global health threat [8].

The prescription of antimicrobial medications constitutes a fundamental component of medical practice, necessitating meticulous consideration and disciplinary knowledge. The process entails a selection of an appropriate antimicrobial agent contingent upon the specific type of infection, the suspected or confirmed causative microorganism, and the individual characteristics of the patient, including age, renal function, and allergy history. Accurate diagnosis and susceptibility testing are essential to confirm the efficacy of the selected antimicrobial agent against the pathogen [9]. Furthermore, it is essential to carefully ascertain the dosage, route of administration, and duration of treatment to optimize efficacy while concurrently reducing the likelihood of adverse effects and the emergence of resistance. Healthcare providers are required to inform patients regarding the significance of adhering to the prescribed treatment plan, the possible adverse effects, and the risks associated with misuse, including the omission of doses or the utilization of surplus antibiotics. Adherence to appropriate antimicrobial guidelines is crucial for maintaining the efficacy of these essential medications, mitigating the prevalence of antimicrobial resistance, and guaranteeing optimal patient outcomes [10].

The widespread administration of antibiotics in the absence of a prescription or appropriate medical oversight may facilitate the emergence of multidrug-resistant pathogens. Antimicrobial agents serve as essential tools in the management and prophylaxis of infectious diseases. Their appropriate utilization, in conjunction with ongoing research and development, is essential to maintain their efficacy and tackle the growing issue of antimicrobial resistance.

As healthcare professionals, pharmacists in Jordan and other surrounding countries play an integral role in antibiotic over-the-counter prescriptions [11,12,13]. This attitude exaggerates the resistance against antibiotics globally, especially in the case of an inappropriate need for antibiotics, self-medication, and consumption behaviors [14]. This impactful problem fires the alarm to face another suspected multidrug-resistant bacterial pandemic, especially after the COVID-19 pandemic [15,16]. In this study, we investigated over-the-counter prescriptions of antibiotics in Jordan, in addition to exploring pharmacists’ antibiotic selections according to their practices. Here, we evaluate the source of information and confidence of prescribing antimicrobials, the type of prescribed antimicrobials, the occurrences of requesting non-prescribed antimicrobials, the presence of challenges facing patients seeking antimicrobials without prescriptions, and pharmacists’ responses when they face patients without prescriptions. Consequently, it is essential to tackle the inappropriate use of antimicrobial agents alongside the insufficient understanding of their function in Jordan, with the objective of enhancing knowledge and practices related to antibiotic utilization among pharmacists and patients [17].

## 2. Results

### 2.1. Study Participants

This investigation revealed that the number of female pharmacists surpassed that of their male counterparts, with 141 and 103 participants, respectively. Furthermore, the age distribution of pharmacists indicated that the predominant group falls within the 18–35 year age range, with a significant concentration of individuals under the age of 35. Participants’ professional experiences were categorized into four groups: less than 5 years, 5–10 years, 11–20 years, and more than 20 years.

Regarding the positions held during the survey period, participants were identified as training pharmacists, pharmacists, experienced pharmacists, senior pharmacists, and pharmacy managers. A table below demonstrates detailed descriptive data of the participants (Table 1).

### 2.2. Prescribing Antimicrobials

#### 2.2.1. Pharmacists’ Confidence of Antimicrobial Prescriptions

**Figure 1:**
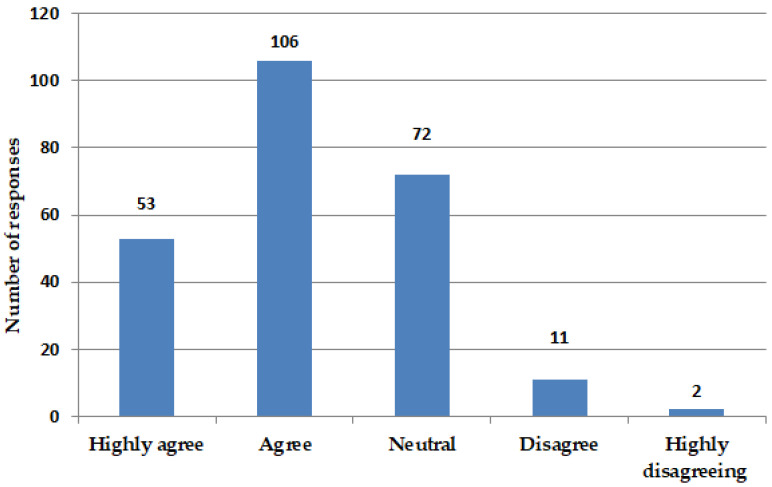
Confidence of prescribing antimicrobials. Figure illustrates level of confidence among pharmacists regarding prescription of antimicrobials. Total number of responses was 244, with 53 expressing “highly agree”, 106 “agree”, 72 “neutral”, 11 “disagree”, and 2 “highly disagreeing”. Question posed was, “I feel confident to assess the appropriateness of antimicrobial prescriptions”.

Pharmacists demonstrate a high level of confidence in assessing the appropriateness of prescribing antimicrobial agents. A notable percentage of pharmacists, specifically those who either agree (106 out of 244, 43.4%) or strongly agree (53 out of 244, 21.7%), demonstrate confidence in their ability to assess the appropriateness of antimicrobial prescriptions. A subset of pharmacists, comprising 72 individuals from a total of 244 (29.5%), exhibits a neutral stance in their responses. A total of 13 pharmacists either expressed disagreement or strong disagreement with the statement, suggesting a deficiency in confidence regarding the prescription of antimicrobials (Figure 1). Significance statistical variations in confidence appear based on the pharmacists’ positions (*p*-value = 0.01) (Table S2).

#### 2.2.2. Role of Resources in Prescribing Antimicrobials

A range of resources was utilized to assist pharmacists in prescribing antimicrobials. This research evaluated the utilization of various resources by pharmacists, including decision support software (Lexicomp, MedscapePage: 4), educational materials and trainings, local antimicrobial resistance data, and clinical practice guidelines.

**Figure 2:**
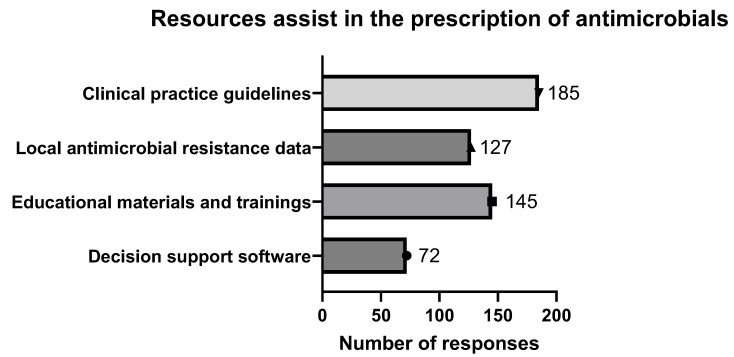
Resources assist in prescription of antimicrobials. Various resources were employed to aid pharmacists in making decisions regarding prescription of antimicrobials. Figure illustrates prevalence of use of these resources among pharmacists in Jordan. Question posed was, “What resources or tools do you find helpful in supporting your decision-making process for antimicrobial prescriptions? (Select all that apply)”.

Pharmacists were permitted to select more than one option for this type of question, resulting in a total of 529 responses. The most frequently used resource among pharmacists was the clinical practice guidelines (35%). Conversely, the least used resource was decision support software (14%). These findings suggest that pharmacists rely on multiple resources to enhance their capability in prescribing antimicrobials (Figure 2). Out of all responses, only two pharmacists did not respond to the question of source of assistant; detailed pharmacists’ responses are shown in Table 2.

#### 2.2.3. Types of Prescribed Antimicrobials

**Figure 3:**
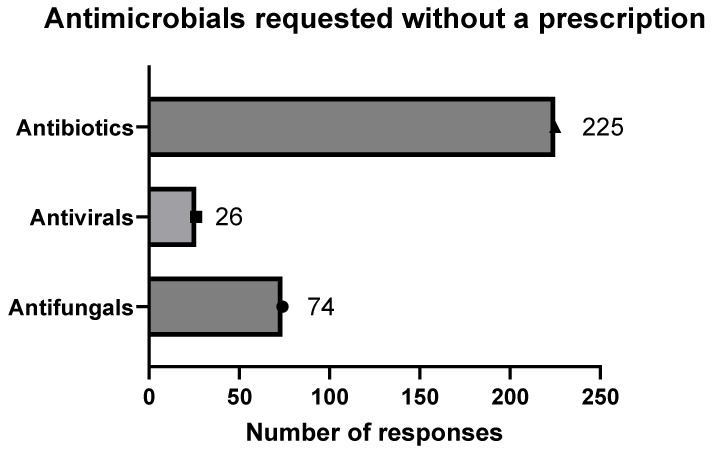
Types of antimicrobials requested without prescription. Pharmacists’ responses to patients’ requests for antimicrobials without prescription. Query was, “What types of antimicrobials are most frequently requested without a prescription? (Select all that apply)”.

Community pharmacies are subject to less stringent oversight regarding antimicrobial prescriptions, resulting in instances where patients obtain antimicrobials without proper prescriptions. In this study, we examined specific antimicrobial prescriptions that patients seek. Pharmacists were allowed to select numerous choices for the sorts of antimicrobials asked about by patients. There were 325 responses to the available choices, which included antibiotics, antifungals, and antivirals. Antibiotics were the most often requested antimicrobials without a prescription, accounting for 225 out of 325 replies (69%), followed by antifungals (74 out of 325 responses, 23%) and antivirals (26 out of 325 responses, 8%) (Figure 3).

#### 2.2.4. Frequently Used Antibiotics

The most common infections that prompt patients to seek antibiotics are throat infection, urinary tract infection, chest infection, and otitis media. In this study, pharmacists were asked about the most often given antibiotics for various infections, and missing responses were not discussed, yielding a total of 220 pharmacists. Penicillin is the primary antibiotic for treating throat infections, prescribed by 75% of pharmacists, while cephalosporin generations are preferred by fewer than 17%. Fluoroquinolones represent the most frequently prescribed antibiotics for urinary tract infections, comprising 48.2% of prescriptions, while cephalosporin generations follow as the second most common option at 35.9%. This indicates that Fluoroquinolones and cephalosporins are the main treatments for urinary tract infections. In the cases of chest infections, penicillins are the most commonly prescribed antibiotics, accounting for 70.1% of prescriptions, while cephalosporin generations represent 25.9%. Beta-lactams are recognized as the optimal treatment for chest infections. Otitis media presents various treatment modalities, with Macrolides as the predominant choice at 43.2%, succeeded by penicillins at 21.8%, cephalosporins at 21.8%, and Fluoroquinolones at 11.8% (Table 3).

### 2.3. The Misuse of Antimicrobials

#### 2.3.1. Pharmacists Struggle with Over-the-Counter Antibiotic Prescriptions

In Jordan, individuals are allowed to obtain antimicrobials without prescriptions. However, this study reveals that there are instances where individuals seek medical prescriptions for antimicrobials without adequate justification or a proper diagnosis to acquire the medications.

**Figure 4:**
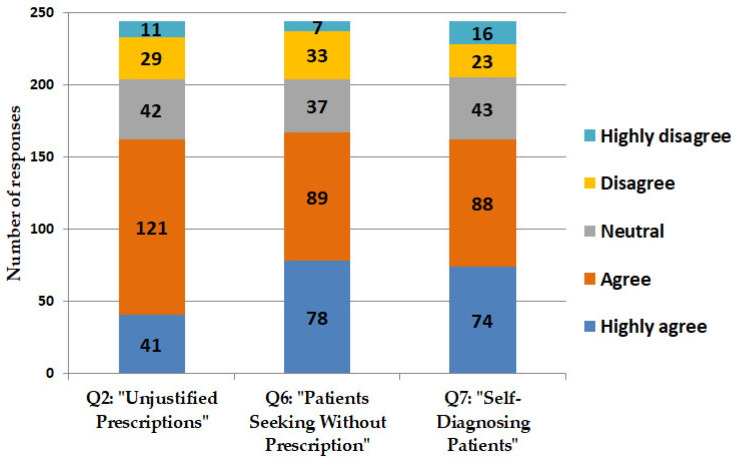
Pharmacists’ responses regarding prescribing antimicrobials without formal prescriptions. Figure presents responses provided by pharmacists regarding their interactions with patients who request antimicrobials without prescriptions in Jordan. Questions encompassed: Q2: “I frequently receive prescriptions for antimicrobials without proper justification or diagnosis.” Q6: “I frequently encounter patients seeking to purchase antimicrobials without a prescription.” Q7: “I frequently encounter patients who self-diagnose and request specific antimicrobials without a prescription”.

Professionals often come across individuals who do not have prescriptions, adequate justification, or a proper diagnosis, or who attempt self-diagnosis while looking to acquire antimicrobials. The results indicate a notable inappropriate use of antimicrobials within the Jordanian community (Figure 4).

Pharmacists frequently face challenges when addressing requests for antimicrobials that lack accompanying prescriptions. Their responses to enquiries concerning these challenges are presented, which encompass the acquisition of requisite patient information for the appropriate prescription of antimicrobials and the regulatory or legal dilemmas encountered when declining to dispense antimicrobials.

**Figure 5:**
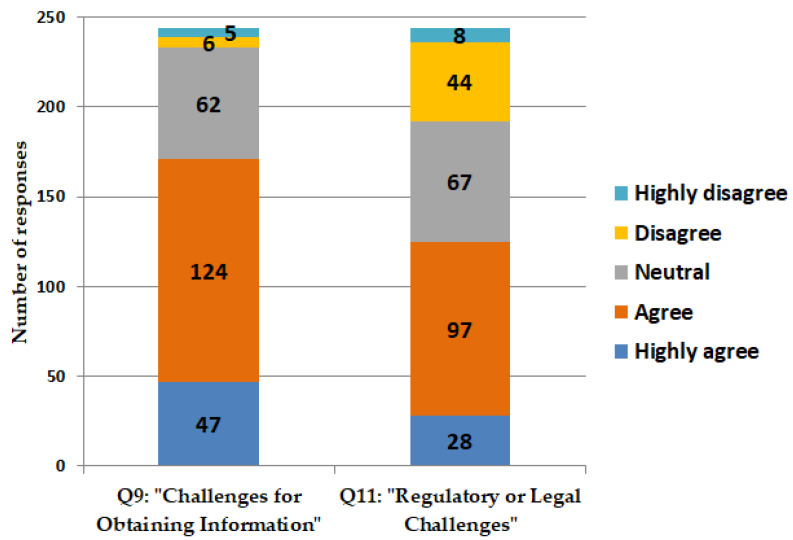
Challenges encountered by scientists in prescribing antimicrobials. Enquiries are delineated as follows: Q9: “I frequently encounter challenges in obtaining relevant patient information necessary for assessing the appropriateness of antimicrobial prescriptions.” Q11: “I frequently encounter regulatory or legal challenges when I refuse to provide antimicrobials to patients seeking to purchase them without a prescription”.

Large proportions of pharmacists either concur or strongly concur that they face difficulties in acquiring relevant information necessary for the appropriate prescription of antimicrobials. A considerable proportion of pharmacists indicate concurrence regarding the challenges encountered when denying the sale of antimicrobials in the absence of prescriptions. Such refusals may result in regulatory or legal challenges for pharmacists (Figure 5).

#### 2.3.2. Actions Taken by Pharmacists When Patients Request Antimicrobials Without Prescription

**Figure 6:**
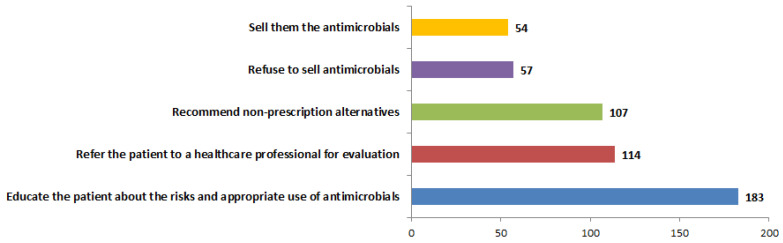
Actions undertaken by pharmacists in response to patient requests for antimicrobials in absence of prescriptions. Responses of pharmacists to patients requesting antimicrobials without prescriptions were examined. This study examines prevalent practices as reported by experienced pharmacists in Jordan. Question posed was, “What actions do you typically take when a patient requests to purchase antimicrobials without a prescription? (Select all that apply)”.

The regulation of antimicrobial prescriptions in private pharmacies is comparatively less stringent than that in hospitals, highlighting the critical function of pharmacists in managing antimicrobial prescriptions. This research assessed the measures implemented by scientists in response to patients requesting antimicrobials without a valid prescription. The survey presented pharmacists with several options, including the sale of antimicrobials, refusal of the sale, referral of the patient to a healthcare professional, recommendation of non-prescription alternatives, and provision of education to the patient regarding the risks and appropriate use of antimicrobials. A significant proportion of pharmacists (36%) indicated a preference for providing education to patients regarding the risks linked to the use of antimicrobials without prescriptions, as well as the appropriate utilization of these medications. A subset of the responses (11%) demonstrated that pharmacists would decline to dispense antimicrobials to patients in the absence of prescriptions. Furthermore, the referral of patients for professional evaluation and the suggestion of non-prescription alternatives were chosen by comparable proportions of respondents, with 114 (22%) and 107 (21%), respectively. A subset of responses indicated that 54 out of 515 participants (10%) expressed a willingness among pharmacists to dispense antimicrobials to patients without the requirement of prescriptions (Figure 6).

## 3. Discussion

Antimicrobial resistance represents a growing public health challenge on a global scale. Pharmacists assume a critical function in the prescription of antimicrobials, attributable to their specialized knowledge in medication management and their availability to patients. A cross-sectional survey conducted among community scientists assessed their confidence levels in prescribing antimicrobials and the resources utilized to inform their prescribing decisions (159 out of 244, Figure 1). A majority of pharmacists indicated a high level of confidence in the prescribing of antimicrobials. The resources most commonly utilized comprised clinical practice guidelines, as well as educational materials and training (Figure 2). Pharmacists are strategically positioned to enhance antimicrobial stewardship through confident prescribing practices that are underpinned by dependable resources. Consistent participation in professional development and access to current clinical guidelines are essential for sustaining effective prescribing practices. The confidence of pharmacists in antimicrobial prescribing, coupled with the utilization of appropriate resources, significantly strengthens the efficacy of antimicrobial prescribing practices. It is imperative that pharmacists are provided with access to reliable information and continuous training to maintain effective prescribing practices and address the issue of antimicrobial resistance.

A significant proportion of pharmacists indicated that they often encounter patients who seek antimicrobials without prescriptions or rely on self-diagnosis for their requests. The aforementioned situations frequently result in the inappropriate use of antimicrobials, thereby exacerbating the growing issue of resistance. Furthermore, pharmacists encounter difficulties associated with incomplete medical histories and regulatory limitations, which further exacerbate the complexities of appropriate antimicrobial prescribing. The identified issues highlight the necessity for improved regulatory frameworks and educational initiatives aimed at bolstering pharmacists’ capacities in the prescribing of antimicrobials. There exists a pressing necessity for the implementation of policies aimed at dissuading self-medication and non-prescription requests, alongside the establishment of systems that guarantee pharmacists have access to comprehensive patient medical histories. It is imperative to address these challenges to mitigate antimicrobial misuse and combat resistance effectively. Accurate information is critical for the appropriate prescription of antimicrobials. Furthermore, a comprehensive understanding of patients’ medical histories is essential for the evaluation of their cases. The refusal to dispense antimicrobials presents challenges for pharmacists, representing considerable obstacles in the prescription process.

The non-prescription use of antimicrobial drugs, particularly antibiotics, raises significant concerns due to its potential contribution to the promotion of antimicrobial resistance. The use of ineffective pharmaceuticals or inappropriate dosages may elevate the likelihood of selecting prescriptions for resistant organisms that are challenging to eradicate [16,17].

The inappropriate use of antimicrobial agents has emerged as a significant global concern. A cross-sectional study conducted in Nigeria revealed that 60.7% (*n* = 509) of consumers held the belief that antibiotics are effective against all infections, 57.4% lacked awareness regarding antibiotic resistance, and 72.5% reported having used antibiotics within the preceding 12 months [18].

In a comparable investigation conducted in Pakistan, it was found that 39.2% (*n* = 196) of participants had engaged in self-medication with antibiotics within the preceding six months, while 42% (*n* = 207) exhibited non-adherence to their prescribed antibiotic treatment regimen [19].

In Europe, despite the imposition of restrictions on non-prescriptive antibiotics, 50% (*n* = 8221) of individuals reported having taken antibiotics within the preceding 12 months. Among these, 22% acquired antibiotics without a prescription, while 8% utilized leftover antibiotics. Furthermore, 84% of participants demonstrated a deficiency in understanding the proper use of antibiotics [20].

A recent cross-sectional study conducted in the UAE revealed that 25.51% (*n* = 1074) of participants had utilized antibiotics within the past 6 months, 24.21% within the past 12 months, and 19.18% had done so more than 12 months ago. Among the subjects surveyed, a mere 12% reported the use of non-prescribed antibiotics. Furthermore, 50% ceased treatment upon completion of the prescribed course, while 22.81% discontinued use when symptoms resolved. Additionally, 47.02% indicated that they stored unused antibiotics for future use, and 7.73% admitted to sharing these medications with family members [21].

Clinical guidelines indicate that penicillin should be considered the primary treatment option for otitis media [22]. It was observed that certain pharmacists opt to prescribe Macrolides as an alternative. In a comparable manner, established guidelines recommend the use of sulphonamides for the treatment of urinary tract infections (UTIs) [23]; however, a subset of pharmacists choose to prescribe Fluoroquinolones instead. Such deviations may result in the misuse of antimicrobials, thereby fostering resistance and leading to less than optimal patient outcomes. The observed deviations can be attributed to potential miseducation or insufficient awareness of the current guidelines among pharmacists. Notwithstanding the existence of established guidelines, certain pharmacists may lack adequate familiarity with the current recommendations. The inappropriate use of antimicrobials resulting from non-adherence to clinical guidelines represents a significant concern that exacerbates the problem of antimicrobial resistance. By addressing the fundamental causes, including inadequate education and insufficient awareness, and by implementing targeted interventions, the prescribing practices of pharmacists can be harmonized with established clinical guidelines. The alignment in question is critical for the optimization of patient outcomes, the reduction in resistance, and the assurance of the effective utilization of antimicrobial agents.

Systematic protocols monitoring the prescribing and administrating of antimicrobials were approached and called “Antimicrobial stewardship” [24]. The aim of this approach is to support and educate professionals in healthcare settings. Educated healthcare professionals will monitor the effectiveness of antimicrobial use. The effectiveness of antimicrobials use will improve patients’ outcomes and reduce the occurrence of antimicrobial resistance [25]. In this investigation, the antimicrobial use indicated that antimicrobial stewardship must be followed to reduce antimicrobial resistance outcomes.

## 4. Materials and Methods

### 4.1. Sampling

Four months of filling the survey were carried out between 7 September 2023 and 6 January 2024. A cohort of community pharmacists participated in this survey, comprising 244 individuals exclusively employed in private (community) pharmacies, and completed the 37-question survey online using Google forms (Table S1). The survey was carefully designed in the Google form platform, then a QR code of the survey link was generated. The survey was distributed among pharmacists by sending the QR code to their work groups on different social media such as Whatsapp or Facebook. Also, the survey was distributed by direct request to fill the survey by QR code to working pharmacists in the community pharmacies. The survey was conducted on working pharmacists in Jordan.

### 4.2. Development and Description of Survey Tool

The authors identified a deficiency in the comprehension of antimicrobial prescribing practices, leading them to evaluate the existing conditions of antimicrobial prescribing in Jordan. In order to carry out this assessment, the authors developed a multi-tiered evaluation focusing on five critical domains of information: (1) The sources of information utilized by pharmacists when prescribing antimicrobials and their corresponding level of confidence in this process. (2) The classification and prevalence of prescribed antimicrobials. (3) Encounters involving patients who seek antimicrobials without a prescription. (4) Challenges encountered by pharmacists in the antimicrobial prescribing process. (5) The extent of awareness among pharmacists regarding non-prescription antimicrobial requests and their corresponding responses. The authors formulated a questionnaire aimed at examining antimicrobial prescribing practices in Jordan.

### 4.3. Data Analysis

The demographic characteristics were compared between samples of the population of pharmacists in Jordan. The percentage, mean, and median were calculated for the categorical variables. An independent sample Kruskal–Wallis test was performed for categorical variables. We used IBM SPSS statistic version 25 (SPSS, Chicago, IL, USA) and Microsoft excel for data analysis.

## 5. Conclusions

This study supports that antimicrobial resistance is a critical global health crisis fueled by inappropriate antimicrobial use, non-compliance with clinical guidelines, and self-medication. Pharmacists, key health providers, play a crucial role in addressing antimicrobial resistance through guideline-based prescribing, participation in antimicrobial stewardship programs, and patient education. Conversely, incomplete patient histories, insufficient awareness of guidelines, and regulatory shortcomings should be addressed for education improvement, stricter regulations, and improved access to medical information. Collaborative efforts are necessary to ensure the alignment of the established clinical protocols with prescribing practices, optimizing patient care and effectively tackling antimicrobial resistance.

## Figures and Tables

**Table 1:** Descriptive analysis providing detailed overview of participants in this study.

Categories	Total (%)
Gender	
Male	103 (42.2%)
Female	141 (57.8%)
Age	
18–25 years	69 (28.3%)
26–35 years	87 (35.7%)
36–45 years	42 (17.2%)
more than 45 years	46 (18.9%)
Experience	
Less than 5 years	110 (45.1%)
5–10 years	48 (19.7%)
11–20 years	48 (19.7%)
More than 20 years	38 (15.6%)
Position	
Training pharmacist	27 (11.1%)
Pharmacist	115 (47.1%)
Experienced pharmacist	47 (19.3%)
Senior pharmacist	17 (7.0%)
Pharmacy manager	38 (15.6%)

**Table 2:** The pharmacists’ responses for the resources that assist in the prescription of antimicrobials.

Question Option	Responses
Only “Clinical practice guidelines”	44
Only “Decision support software”	2
Only “Educational materials and trainings”	21
Only “Local antimicrobial resistance data”	18
“Clinical practice guidelines” and “Decision support software”	5
“Clinical practice guidelines” and “Educational materials and trainings”	30
“Clinical practice guidelines” and “Local antimicrobial resistance data”	24
“Decision support software” and “Educational materials and trainings”	5
“Educational materials and trainings” and “Local antimicrobial resistance data”	8
“Clinical practice guidelines”, “Decision support software”, and “Educational materials and trainings”	12
“Clinical practice guidelines”, “Educational materials and trainings”, and “Local antimicrobial resistance data”	25
“Decision support software”, “Educational materials and trainings”, and “Local antimicrobial resistance data”	3
“Clinical practice guidelines”, “Decision support software”, “Educational materials and trainings”, and “Local antimicrobial resistance data”	45

**Table 3:** Pharmacists’ responses for most common antibiotics prescribed for various infections.

	Throat Infection	Urinary Tract Infection	Chest Infection	Otitis Media
1st generation Cephalosporin	16(7.3%)	31(14.1%)	28(12.7%)	15(6.8%)
2nd generation Cephalosporin	7(3.2%)	30(13.6%)	14(6.4%)	17(7.7%)
3rd generation Cephalosporin	13(5.9%)	18(8.2%)	15(6.8%)	16(7.3%)
Fluoroquinolones	2(0.9%)	**106(48.2%)**	2(0.9%)	26(11.8%)
Macrolides	15(6.8%)	6(2.7%)	4(1.8%)	**95(43.2%)**
Penicillins	**165(75%)**	16(7.3%)	**156(70.1%)**	48(21.8%)
Sulphonamides	2(0.9%)	12(5.5%)	1(0.5%)	2(0.9%)
Tetracyclines	0(0%)	1(0.5%)	0(0%)	1(0.5%)

Bold and underlined numbers refer to highest response.

## Data Availability

The questionnaire was provided in Supplement S3.

## Supplementary Materials

The following supporting information can be downloaded at: https://www.mdpi.com/article/10.3390/antibiotics14030300/s1. Table S1: List of questions and participants’ responses. Table S2: Variation in five critical domains of information. Supplement S3: List of questions that were asked for to pharmacists.
