# Supplementary material for: Community Pharmacists’ Responses Toward Antimicrobial Prescriptions in Jordan: A Cross-Sectional Survey
Source: Antibiotics (Basel). 2025 Mar 14;14(3):300. doi: 10.3390/antibiotics14030300 (PMC11939746; doi:10.3390/antibiotics14030300)
Supplement: Supplementary file 1 [file antibiotics-14-00300-s001.zip › antibiotics-3399034-supplementary.pdf]

Table S1: List of questions and participants' responses

Questions	N	Highly Disagree	Disagree	Neutral	Agree	Highly Agree	Mean (Sd)	Median (Q1 - Q3)
1. I frequently encounter patients seeking antimicrobial medication	244	n= 5 (2.1%)	n= 12 (4.9%)	n= 53 (21.7%)	n= 102 (41.8%)	n= 72 (29.5%)	3.92 (0.944)	4 (3 - 5)
2. I frequently receive prescriptions for antimicrobials without proper justification or diagnosis	244	n= 11 (4.5%)	n= 29 (11.9%)	n= 42 (17.2%)	n= 121 (49.6%)	n= 41 (16.8%)	3.62 (1.041)	4 (3 - 4)
3. I feel confident to assess the appropriateness of antimicrobial prescriptions	244	n= 2 (0.8%)	n= 11 (4.5%)	n= 72 (29.5%)	n= 106 (43.5%)	n= 53 (21.7%)	3.81 (0.856)	4 (3 - 4)
4. I usually communicate with prescribers to clarify or discuss antimicrobial prescriptions	244	n= 7 (2.9%)	n= 42 (17.2%)	n= 67 (27.5%)	n= 93 (38.1%)	n= 35 (14.3%)	3.44 (1.027)	4 (3 - 4)
5. I satisfy the current antimicrobial prescription practices in my pharmacy or healthcare setting	244	n= 5 (2%)	n= 31 (12.7%)	n= 56 (23%)	n= 113 (46.3%)	n= 39 (16%)	3.61 (0.968)	4 (3 - 4)
6. I frequently encounter patients seeking to purchase antimicrobials without a prescription	244	n= 7 (2.9%)	n= 33 (13.5%)	n= 37 (15.2%)	n= 89 (36.4%)	n= 78 (32%)	3.81 (1.114)	4 (3 - 5)
7. I frequently encounter patients who self-diagnose and request specific antimicrobials without a prescription	244	n= 16 (6.6%)	n= 23 (9.4%)	n= 43 (17.6%)	n= 88 (36.1%)	n= 74 (30.3%)	3.74 (1.177)	4 (3 - 5)
8. I am highly concerned about the misuse of antimicrobials without a prescription in my community	244	n= 3 (1.2%)	n= 6 (2.5%)	n= 28 (11.5%)	n= 105 (43%)	n= 102 (41.8%)	4.22 (0.835)	4 (4 - 5)
9. I frequently encounter challenges in obtaining relevant patient information necessary for assessing the appropriateness of antimicrobial prescriptions	244	n= 5 (2%)	n= 6 (2.5%)	n= 62 (25.4%)	n= 124 (50.8%)	n= 47 (19.3%)	3.83 (0.838)	4 (3 - 4)
10. I noticed adverse effects or complications in patients who use antimicrobials without a prescription	244	n= 8 (3.3%)	n= 19 (7.8%)	n= 64 (26.2%)	n= 108 (44.3%)	n= 45 (18.4%)	3.67 (0.973)	4 (3 - 4)

11. I frequently encountered regulatory or legal challenges when I refuse to give antimicrobials to patients seeking to purchase antimicrobials without a prescription	244	n= 8 (3.3%)	n= 44 (18%)	n= 67 (27.4%)	n= 97 (39.8%)	n= 28 (11.5%)	3.38 (1.013)	4 (3 - 4)	
12. I am highly interested in participating in educational programs or campaigns to raise awareness about the appropriate use of antimicrobials	244	n= 1 (0.4%)	n= 5 (2%)	n= 38 (15.6%)	n= 99 (40.6%)	n= 101 (41.4%)	4.2 (0.806)	4 (4 - 5)	
		Chest infection	Otitis media	Throat infection	Urinary tract infection				
18. Which infection most frequently leads patients to ask you for an antimicrobial without prescription? [1st]	232	31	14	145	42				
19. Which infection most frequently leads patients to ask you for an antimicrobial without prescription? [2nd]	212	55	50	38	69				
20. Which infection most frequently leads patients to ask you for an antimicrobial without prescription? [3rd]	205	57	55	31	62				
21. Which infection most frequently leads patients to ask you for an antimicrobial without prescription? [4th]	197	47	72	26	52				
		1st generation Cephalosporin	2nd generation Cephalosporin	3rd generation Cephalosporin	Fluoroquinolones	Macrolides	Penicillins	Sulphonamides	Tetracyclines
22. What is the most common antimicrobial prescribed for throat infection? [1st]	229	22	8	13	2	15	167	2	0
23. What is the most common antimicrobial prescribed for throat infection? [2nd]	200	77	39	25	4	26	28	0	1
24. What is the most common antimicrobial prescribed for throat infection? [3rd]	190	26	70	30	12	30	20	0	2
25. What is the most common antimicrobial prescribed for throat infection? [4th]	171	20	28	31	24	35	23	3	7

26. What is the most common antimicrobial prescribed for urinary tract infection? [1st]	222	33	33	18	102	6	17	12	1
27. What is the most common antimicrobial prescribed for urinary tract infection? [2nd]	188	32	41	34	22	12	15	25	7
28. What is the most common antimicrobial prescribed for urinary tract infection? [3rd]	168	20	47	32	18	9	26	14	2
29. What is the most common antimicrobial prescribed for urinary tract infection? [4th]	157	34	23	19	11	7	40	12	11
30. What is the most common antimicrobial prescribed for otitis media? [1st]	218	29	14	16	2	4	152	1	0
31. What is the most common antimicrobial prescribed for otitis media? [2nd]	189	60	39	40	5	11	30	1	3
32. What is the most common antimicrobial prescribed for otitis media? [3rd]	167	25	52	23	9	29	24	2	3
33. What is the most common antimicrobial prescribed for otitis media? [4th]	154	25	22	25	24	27	23	4	4
34. What is the most common antimicrobial prescribed for chest infection? [1st]	217	15	17	16	26	90	50	2	1
35. What is the most common antimicrobial prescribed for chest infection? [2nd]	189	33	23	26	39	34	24	3	7
36. What is the most common antimicrobial prescribed for chest infection? [3rd]	176	30	34	39	24	19	20	4	6
37. What is the most common antimicrobial prescribed for chest infection? [4th]	162	17	40	24	20	11	37	5	8

Table S2: Variation in five critical domains of information

		patients requesting antimicrobials without a prescription (Q2, Q6, Q7)		Confidence (Q3)		Antimicrobial for throat infection (Q22)		Antimicrobial for UTI (Q26)		Antimicrobial for otitis media (Q30)		antimicrobial for chest infection (Q34)		Challenges (Q9, Q11)	
		Mean Rank	p-value	Mean Rank	p-value	Mean Rank	p-value	Mean Rank	p-value	Mean Rank	p-value	Mean Rank	p-value	Mean Rank	p-value
Gender	Male	133.76	**0.032**	133.66	0.025	116.82	0.646	119.44	0.095	113.85	0.278	111.74	0.564	119.63	0.577
	Female	114.28		114.35		113.64		105.67		106.26		106.98		124.6	
Age	18-25 years	131.67	0.609	111.59	0.238	116.47	0.937	117.33	0.625	118.19	**0.026**	112.38	0.516	113.63	0.461
	26-35 years	120.55		129.07		115.05		108.16		116.18		100.91		126.05	
	36-45 years	119.39		133.36		110.48		116.64		99.51		113.36		133.51	
	more than 45 years	115.27		116.51		116.9		103.75		91.78		115.53		119.03	
Experience	Less than 5 years	125.3	0.884	121.25	0.498	113.78	0.642	115.83	0.758	115.14	**0.03**	111.83	0.327	116.76	0.704
	5-10 years	119.19		125.13		123.39		106.96		116.33		105.78		127.8	
	11-20 years	124.55		132.2		109.52		111.27		108.23		117.47		127.17	
	More than 20 years	116		110.55		116.08		104.81		86.71		93.59		126.53	
Position	Training pharmacist	141.48	0.228	99.65	**0.01**	103.6	**0.047**	134.22	0.402	105.13	0.16	114.67	0.919	99.59	0.303
	Pharmacist	113.45		118.79		117.9		108.72		115.69		108		121.27	
	Experienced pharmacist	133.66		151.69		119.92		105.91		112.02		113.68		131.16	
	Senior pharmacist	132.85		122.79		79.6		118		82.13		108.87		118.35	
	Pharmacy manager	117.97		113.72		122.4		109.24		103.26		102.3		133.66	

Nonparametric independent sample test; Kruskal-Wallis test ($p<0.05$)

S3: List of questions that have been asked for pharmacists.

Gender:

1. Male
2. Female

Age:

1. 25-34
2. 35-44
3. 45-54
4. 55 or above

Years of Experience as a Pharmacist:

1. Less than 5 years
2. 5-10 years
3. 11-20 years
4. More than 20 years

Your work position at pharmacy:

1. Training pharmacist
2. Pharmacist
3. Experienced pharmacist
4. Senior pharmacist
5. Pharmacy manager

		Highly Agree	Agree	Neutral	Disagree	Highly disagree
Q01	I frequently encounter patients seeking antimicrobial medication?					
Q02	I frequently receive prescriptions for antimicrobials without proper justification or diagnosis?					
Q03	I feel confident to assess the appropriateness of antimicrobial prescriptions?					
Q04	I usually communicate with prescribers to clarify or discuss antimicrobial prescriptions?					
Q05	I satisfy the current antimicrobial prescription practices in my pharmacy or healthcare setting?					
Q06	I frequently encounter patients seeking to purchase antimicrobials without a prescription?					
Q07	I frequently encounter patients who self-diagnose and request specific antimicrobials without a prescription?					
Q08	I am highly concerned about the misuse of antimicrobials without a prescription in my community?					
Q09	I frequently encounter challenges in obtaining relevant patient information necessary for assessing the appropriateness of antimicrobial prescriptions?					
Q10	I noticed adverse effects or complications in patients who use antimicrobials without a prescription?					
Q11	I frequently encountered regulatory or legal challenges when I refuse to give antimicrobials to patients seeking to purchase antimicrobials without a prescription?					
Q12	I am highly interested in participating in educational programs or campaigns to raise awareness about the appropriate use of antimicrobials?					

Q13: What factor do you consider when evaluating the appropriateness of an antimicrobial prescription? (Select all that apply)

1. Patient's symptoms
2. Patient's medical history
3. Results of diagnostic tests

4. Clinical guidelines and protocols
5. Local antimicrobial resistance patterns

Q14: What resources or tools do you find helpful in supporting your decision-making process for antimicrobial prescriptions? (Select all that apply)

1. Clinical practice guidelines
2. Antimicrobial stewardship programs
3. Local antimicrobial resistance data
4. Educational materials and trainings
5. Decision support software

Q15: What types of antimicrobials are most commonly requested without a prescription? (Select all that apply)

1. Antibiotics
2. Antifungals
3. Antivirals

Q16: What actions do you typically take when a patient requests to purchase antimicrobials without a prescription? (Select all that apply)

1. Refuse the sale\ Refuse to sell
2. Educate the patient about the risks and appropriate use of antimicrobials
3. Recommend non-prescription alternatives
4. Refer the patient to a healthcare professional for evaluation
5. Sell them the antimicrobials

Q17: In your opinion, what are the main reasons patients seek to obtain antimicrobials without prescriptions? (Select all that apply)

1. Lack of access to healthcare services
2. Self-medication culture
3. Fear of seeking medical attention
4. Cost of healthcare
5. Lack of awareness about the dangers of antimicrobial misuse

		Urinary tract infection	Throat infection	Otitis media	Chest infection
Q18	What is the most common infection that requested for antimicrobials?				
Q19	What is the 2nd most common infection that requested for antimicrobials?				
Q20	What is the 3rd most common infection that requested for antimicrobials?				
Q21	What is the 4th most common infection that requested for antimicrobials?				

		Penicillins.	1st generation Cephalosporin.	2nd generation Cephalosporin.	3rd generation Cephalosporin.	Tetracyclines.	Macrolides.	Fluoroquinolones.	Sulphonamides.
Q22	What is the most common antimicrobial prescribed for throat infection?								
Q23	What is the 2nd most common antimicrobial prescribed for throat infection?								
Q24	What is the 3rd most common antimicrobial prescribed for throat infection?								
Q25	What is the most common antimicrobial prescribed for urinary tract infection?								
Q26	What is the 2nd most common antimicrobial prescribed for urinary tract infection?								
Q27	What is the 3rd most common antimicrobial prescribed for urinary tract infection?								
Q28	What is the most common antimicrobial prescribed for otitis media?								
Q29	What is the 2nd most common antimicrobial prescribed for otitis media?								
Q30	What is the 3rd most common antimicrobial prescribed for otitis media?								
Q31	What is the most common antimicrobial prescribed for chest infection?								
Q32	What is the 2nd most common antimicrobial prescribed for chest infection?								
Q33	What is the 3rd most common antimicrobial prescribed for chest infection?								
